# Comprehensive analysis of differentially expressed miRNAs in mice with kidney injury induced by chronic intermittent hypoxia

**DOI:** 10.3389/fgene.2022.918728

**Published:** 2022-11-01

**Authors:** Yunan Su, Chaowei Li, Weifeng Liu, Yibin Liu, Liangyi Li, Qingshi Chen

**Affiliations:** ^1^ Department of Emergency, The Second Affiliated Hospital of Fujian Medical University, Quanzhou, China; ^2^ Department of Gastroenterology, The Second Affiliated Hospital of Fujian Medical University, Quanzhou, China; ^3^ Department of Anesthesiology, The Second Affiliated Hospital of Fujian Medical University, Quanzhou, China; ^4^ Department of Endocrinology and Metabolism, The Second Affiliated Hospital of Fujian Medical University, Quanzhou, China

**Keywords:** miRNA, microRNA, chronic intermittent hypoxia (CIH), kidney injury, miRNA sequencing, bioinformatics analysis

## Abstract

**Background:** miRNAs have been reported to participate in various diseases. Nevertheless, the expression patterns of miRNA in obstructive sleep apnea (OSA)-induced kidney injury remain poorly characterized. In the current study, miRNA sequencing (miRNA-seq) was conducted to investigate miRNA expression profiles in a chronic intermittent hypoxia (CIH)-induced renal injury mouse model.

**Methods:** The mouse model of chronic intermittent hypoxia was established. Differentially expressed miRNAs (DEmiRs) were detected using miRNA-seq technology. The sequencing data were subjected to Gene Ontology (GO) functional enrichment and the Kyoto Encyclopedia of Genes and Genomes (KEGG) pathway analyses using a bioinformatics approach. RT-qPCR was further used to evaluate the sequencing results. Finally, we created a network for clarifying the relationship between the miRNAs and target genes.

**Results:** In total, nine miRNAs were identified to be upregulated and nine to be downregulated in a mouse model of renal injury induced by chronic intermittent hypoxia. The Kyoto Encyclopedia of Genes and Genomes analyses revealed that the Wnt signaling pathway was involved in the development of chronic intermittent hypoxia-induced renal injury. Subsequently, eight DEmiRs, namely, mmu-miR-486b–3p, mmu-miR-215–5p, mmu-miR-212–3p, mmu-miR-344–3p, mmu-miR-181b-1-3p, mmu-miR-467a–3p, mmu-miR-467 d-3p, and mmu-miR-96–5p, showed a similar trend of expression when verified using RT-qPCR. Finally, five selected DEmiRs were used to construct a miRNA–mRNA network.

**Conclusion:** In conclusion, a total of 18 DEmiRs were identified in the mouse model of chronic intermittent hypoxia-induced renal injury. These findings advance our understanding of the molecular regulatory mechanisms underlying the pathophysiology of obstructive sleep apnea-associated chronic kidney disease.

## Introduction

Obstructive sleep apnea (OSA), a breathing disorder, is characterized by chronic intermittent hypoxia (CIH). The prevalence of OSA is about 17% and 34% in middle-aged women and men, respectively (Yeghiazarians et al., 2021). OSA has been linked to numerous comorbidities, including metabolic disease (Light et al., 2018), nonalcoholic fatty liver disease (Mesarwi et al., 2019), cardiovascular diseases (Schütz et al., 2021), and Alzheimer’s disease (Liguori et al., 2021). Although efforts have been undertaken to comprehend the consequences of OSA, there may be more comorbidities or problems associated with OSA than initially expected. Mounting evidence demonstrates that chronic kidney disease (CKD) is a highly prevalent comorbidity of OSA (Nowinski et al., 2020; Badran et al., 2021). Moreover, the prevalence of OSA in CKD patients is significantly higher than that in the general population (Lin et al., 2020). Patients with severe OSA exhibit a relatively high prevalence among people with CKD. Meanwhile, it has further been found that OSA could accelerate the loss of kidney function. To date, the molecular mechanisms of OSA-associated CKD have remained poorly understood.

miRNAs are small noncoding RNAs that have acted as critical post-transcriptional regulators of gene expression by binding to targeted mRNAs for translational inhibition or destabilization. Hundreds of miRNAs have received substantial attention over the last decades. They play a crucial role in multiple biological processes and pathological events (Pan et al., 2013; Torres et al., 2018; Matsuyama and Suzuki, 2020). For liver diseases, dysregulation of numerous miRNAs is reported to be associated with liver fibrosis, liver injury, and liver metabolism dysregulation (Wang et al., 2021a). In addition, ectopic miR-155–5p expression inhibits radiation-induced epithelium-to-mesenchymal transition *via* the GSK-3β/NF-κB pathway in the pathogenesis of radiation-induced pulmonary fibrosis (Wang et al., 2021b). A recent study demonstrates that miRNAs play an essential role in regulating vascular smooth muscle cells in cardiovascular diseases (Nguyen et al., 2021). However, a comprehensive analysis of the miRNA profile and functions in OSA-induced kidney injury remains to be thoroughly investigated.

In our research, miRNA sequencing (miRNA-seq) was implemented to identify miRNA expression profiles in a mouse model of renal injury induced by OSA. Gene ontology (GO) and Kyoto Encyclopedia of Genes and Genomes (KEGG) analyses were applied to explore the functions of the mRNAs targeted by differentially expressed miRNAs (DEmiRs). In addition, RT-qPCR was further used to verify the sequencing results. Finally, a miRNA–mRNA regulatory network was established. The present study aimed to uncover the potential mechanisms underlying kidney injury in the pathological processes of OSA-related CKD.

## Materials and methods

### Animals

Male balb/c mice were acquired from Beijing Weitong Lihua Experimental Animal Technology Co., Ltd. All experiments were approved by the Institutional Animal Care and Use Committee of the Second Affiliated Hospital of Fujian Medical University.

### Mouse model of chronic intermittent hypoxia

The mice were randomly divided into two groups: the CIH group and the control group. The CIH mouse model was established as described in our previous study (Lai et al., 2021). In brief, the mice were placed in cages with a gas control system, which regulated nitrogen, air, and oxygen flow into the chambers. During the whole period of CIH, the fractional inspired O_2_ was decreased from normal levels (21%) to about 6% within 60 s, followed by reoxygenation to normoxia levels within the next 60 s. The duration of CIH treatment was 8 weeks. The oxygen level was detected with an O_2_ concentration monitor.

### Histopathological examinations

After being euthanized, we collected kidney tissues and fixed them in 4% paraformaldehyde for 24 h. Then, the renal tissues were embedded in paraffin. Subsequently, we performed hematoxylin–eosin (HE) staining on 4 µm thick sections to detect the changes of general cell morphology in kidney tissues. Finally, to make a precise pathological diagnosis, we carefully observed the sections under a light microscope.

### miRNA-seq and bioinformatics analysis

Total RNA was extracted from the injured kidney tissue of mice following CIH exposure. The small RNA libraries were generated with the help of the Illumina TruSeq Rapid SR Cluster Kit. The purified cDNA libraries were utilized for cluster generation on an Illumina’s flow cell and then sequenced on Illumina NextSeq 500 according to the manufacturer’s instructions.

The miRNA deep sequencing data were analyzed by miRDB and miRBase. Volcano plot analysis was conducted using R software. Hierarchical clustering was performed using Cluster 3.0. GO analysis was used to categorize the function of DEmiRs. The KEGG pathway analysis was used to identify the main signaling pathways. The miRNA-seq experiment was employed in triplicate.

### Validation of reverse transcription-quantitative PCR (RT-qPCR)

The expression of DEmiRs was measured by RT-qPCR using an ABI 7500 System. Primer sequences are presented in Table 1. The relative amounts were determined using the 2^−ΔΔCt^ method, with U6 siRNA as the internal control. All RT-qPCR reactions were performed three times.

**TABLE 1:** Primers used for RT-qPCR.

Gene	Sequence (5'->3′)	Length (bp)
U6	F:5′GCTTCGGCAGCACATATACTAAAAT3′	89
	R: 5′CGC​TTC​ACG​AAT​TTG​CGT​GTC​AT3′	
mmu-miR-467a–3p	GSP: 5′GGG​GGG​CAT​ATA​CAT​ACA​CAC​A3′	66
	R: 5′GTG​CGT​GTC​GTG​GAG​TCG3′	
mmu-miR-486b–3p	GSP: 5′GAA​ACG​GGG​CAG​CTC​AGT3′	62
	R: 5′GTG​CGT​GTC​GTG​GAG​TCG3′	
mmu-miR-215–5p	GSP: 5′GGG​GGA​TGA​CCT​ATG​ATT​TG3′	64
	R: 5′GTG​CGT​GTC​GTG​GAG​TCG3′	
mmu-miR-344–3p	GSP: 5′GGG​TGA​TCT​AGC​CAA​AGC​CT3′	64
	R: 5′GTG​CGT​GTC​GTG​GAG​TCG3′	
mmu-miR-181b-1-3p	GSP: 5′GGG​GGC​TCA​CTG​AAC​AAT​G3′	64
	R: 5′GTG​CGT​GTC​GTG​GAG​TCG3′	
mmu-miR-467days-3p	GSP: 5′GGG​GGG​GAT​ACA​TAC​ACA​CAC3′	65
	R: 5′GTG​CGT​GTC​GTG​GAG​TCG3′	
mmu-miR-96–5p	GSP: 5′GGT​TTG​GCA​CTA​GCA​CAT3′	66
	R: 5′CAG​TGC​GTG​TCG​TGG​AGT3′	
mmu-miR-212–3p	GSP:5′GGGGGATAACAGTCTCCAGTCA3′	66
	R:5′GTGCGTGTCGTGGAGTCG3′	

### Prediction of miRNA targets

To reveal the potential functions of these DEmiRs, their predicted target genes were filtered out according to the intersection of the miRDB and TargetScan databases. The miRNA–mRNA interaction network was created and visualized with the use of Cytoscape software.

### Statistical analysis

Data were analyzed using SPSS (version 19.0). They were presented as the mean ± Sd. Comparisons between the two groups were performed using an unpaired Student’s *t*-test. Differences were considered significant at *p* < 0.05.

## Results

### Chronic intermittent hypoxia-induced renal histological damage

**FIGURE 1:**
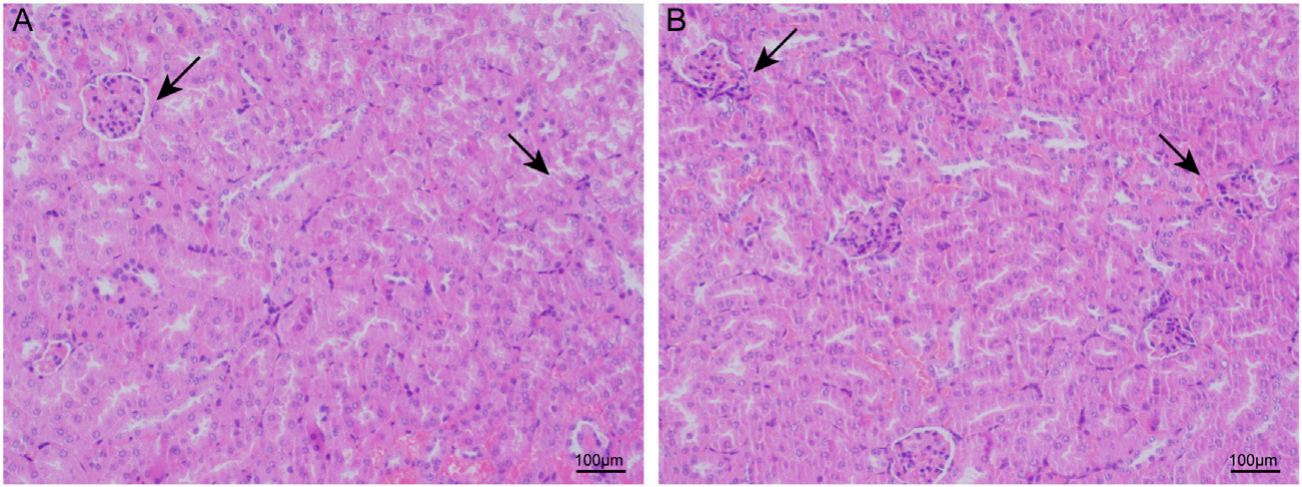
CIH caused renal structure damage in mice. In light microscopic examination, glomerular and renal tubular epithelial cell swelling and glomerular atrophy were more remarkable in the renal tissues of the CIH groups **(B)**.

With HE staining, we found that the glomerular and tubular structures were normal in the control group. Compared to the control groups, the CIH groups had significant changes such as glomerular atrophy, glomerular swelling, and renal tubular epithelial cell swelling (Figure 1).

### Identification of DEmiRs in the renal injury mouse model induced by chronic intermittent hypoxia

**FIGURE 2:**
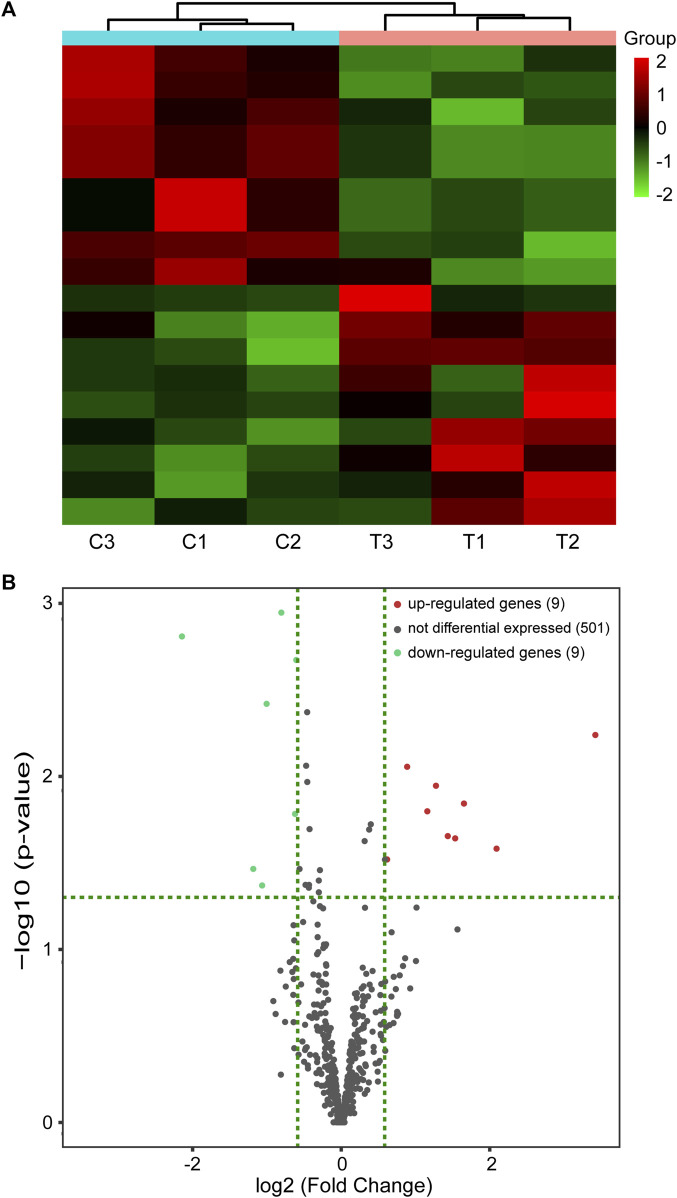
miRNA-seq data corresponding to the DEmiRs. **(A)** Hierarchical cluster of DEmiRs. Green represents downregulation and red represents upregulation. **(B)** Volcano plots. Red and blue dots represent DEmiRs. miRNA-seq, miRNA sequencing and DEmiRs, differentially expressed microRNAs.

miRNA-seq was carried out to validate the differential expression of miRNAs in the renal injury mouse model caused by CIH. In total, 18 DEmiRs were identified in the mouse model of CIH-induced kidney injury, including nine upregulated and nine downregulated DEmiRs. Hierarchical clustering analysis and volcano plot analysis of all differentially expressed miRNAs are displayed in Figures 2A,B, respectively.

### Gene Ontology enrichment analysis

**FIGURE 3:**
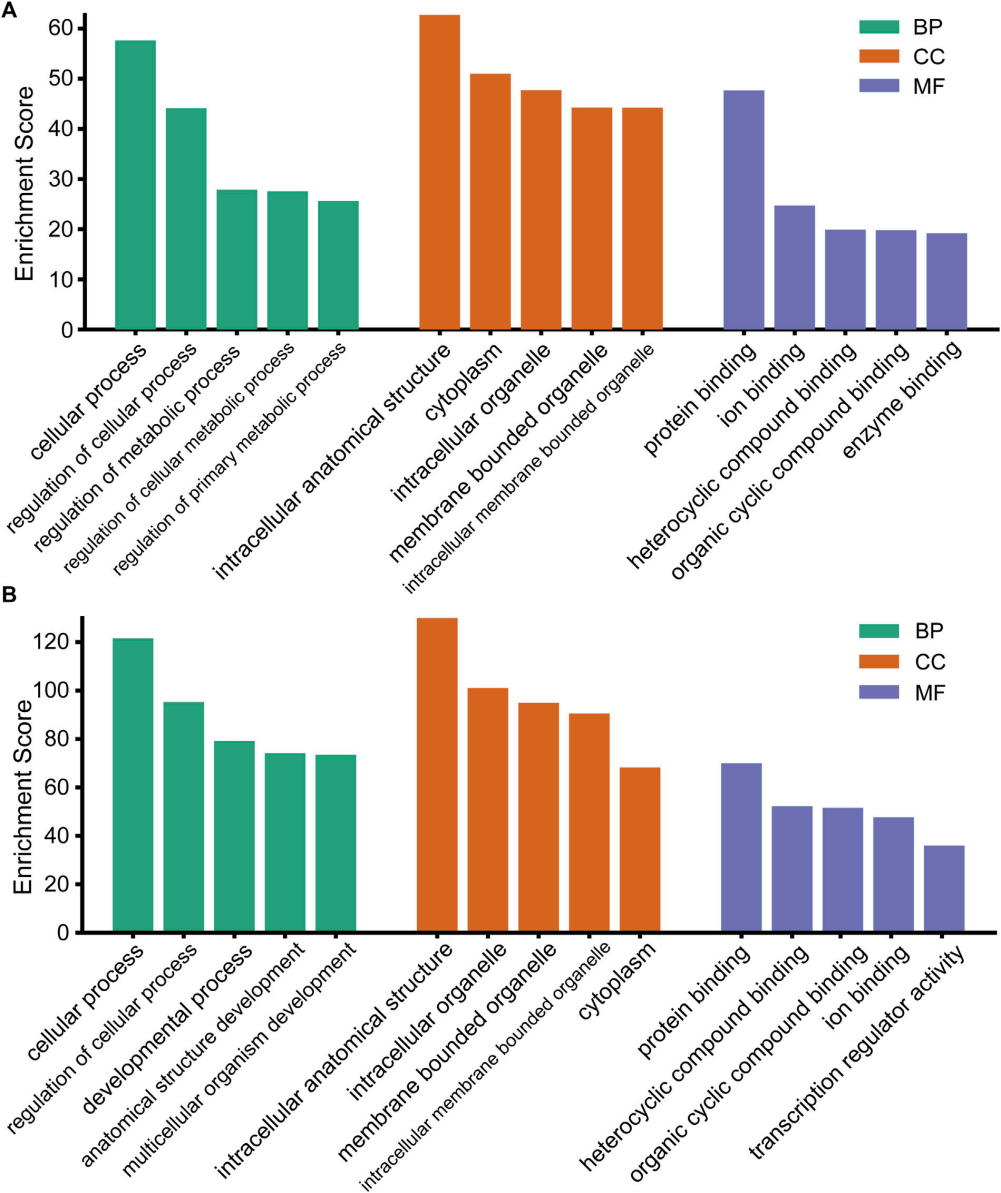
GO analysis of the target genes of DEmiRs. **(A)** GO terms for the upregulated DEmiRs. **(B)** GO terms for the downregulated DEmiRs.

To better reveal the potential roles of these DEmiRs in the etiopathogenesis of CIH-induced kidney injury, the targeted genes were then submitted to the GO database for functional annotation. That is, all the predicted genes were analyzed with their GO annotation, which included cellular components (CCs), molecular functions (MFs), and biological processes (BPs). The results demonstrated that the targeted genes of all DEmiRs were mostly responses to the terms “cellular process” (BP), “intracellular anatomical structure” (CC), and “protein binding” (MF) (Figures 3A,B). This finding indicated that the expression of these DEmiRs was crucial during the occurrence of renal injury caused by CIH.

### Kyoto Encyclopedia of Genes and Genomes enrichment analysis

**FIGURE 4:**
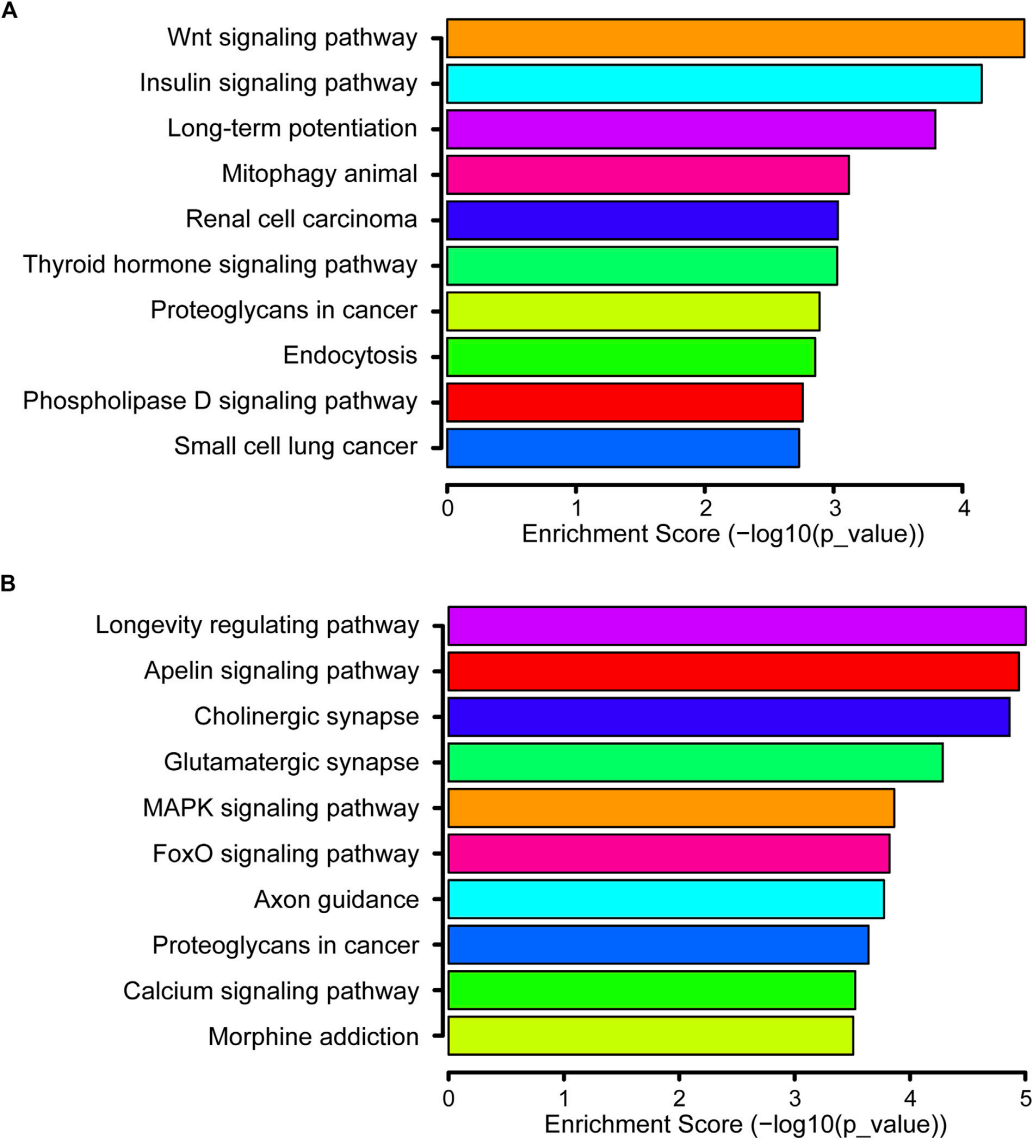
KEGG pathway. **(A)** Top 10 significant pathways associated with upregulated genes are listed. **(B)** Top 10 significant pathways related to downregulated genes are presented.

There were 141 KEGG pathways for all DEmiRs. The top 10 pathways of up- and down-regulated DEmiRs are listed in Figures 4A,B, including the MAPK signaling pathway, Wnt signaling pathway, and the FoxO signaling pathway. The most significantly enriched pathway of downregulated DEmiRs was the Wnt signaling pathway. These results suggested that the annotated pathways were important for the DEmiRs involved in the development of CIH-induced renal injury. In addition, our findings pointed to 61 KEGG pathways for the upregulated and 80 KEGG pathways for the downregulated miRNAs.

### Verification of DEmiRs using RT-qPCR

**FIGURE 5:**
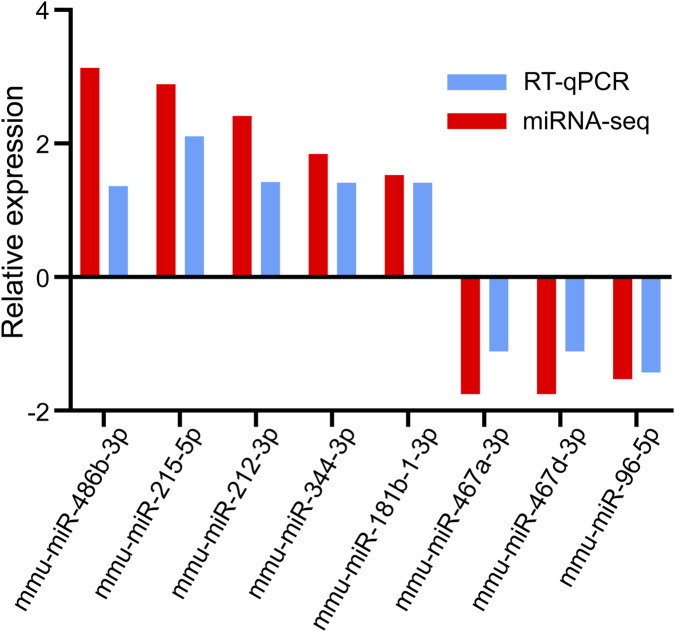
Validation of candidate DEmiRs by RT-qPCR. miRNA-seq, miRNA sequencing.

RT-qPCR was used to determine the expression levels of the eight selected DEmiRs. The results demonstrated that five upregulated miRNAs (mmu-miR-486b–3p, mmu-miR-215–5p, mmu-miR-212–3p, mmu-miR-344–3p, and mmu-miR-181b-1-3p) and three downregulated miRNAs (mmu-miR-467a-3p, mmu-miR-467 d-3p, and mmu-miR-96–5p) identified through miRNA-seq showed a similar trend of expression when verified using RT-qPCR (Figure 5). This finding suggested that our miRNA-seq data were reliable.

### Construction of the miRNA–mRNA network

**FIGURE 6:**
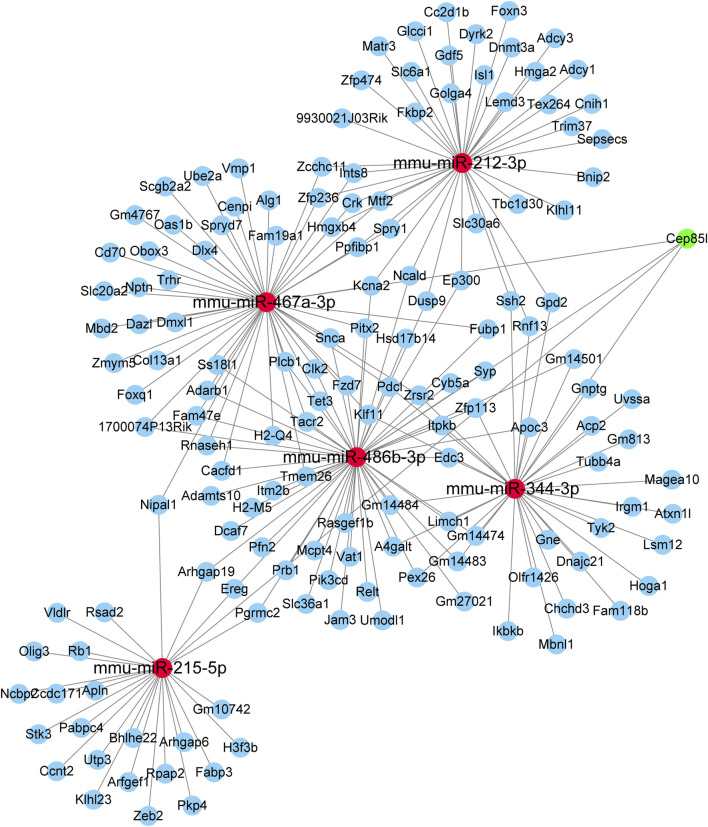
miRNA–mRNA network constructed using five DEmiRs. Red nodes indicate miRNAs. Blue nodes represent mRNAs.

In total, five selected miRNAs, namely, mmu-miR-486b–3p, mmu-miR-344–3p, mmu-miR-215–5p, mmu-miR-212–3p, and mmu-miR-467a-3p, and their targeted genes were assembled in the integrated miRNA–mRNA network. The miRNA–mRNA network generated with the use of the five aforementioned DEmiRs was created using Cytoscape (Figure 6). Our results provided a novel research strategy to explore the underlying mechanism of these DEmiRs by revealing their targeted mRNAs.

## Discussion

To the best of our knowledge, our current study is the first to report a comprehensive analysis of the differential expression profile of miRNAs in kidney tissue of a CIH mouse model. The possible functions and enriched signaling pathways of DEmiRs in CIH-induced renal injury were then systematically analyzed. Specific miRNAs were selected to validate the sequencing data using RT-qPCR. Our findings deepen our understanding of how changes in miRNA expression were involved in the underlying mechanisms of OSA-associated CKD.

OSA has been deemed as one of the leading causes of kidney impairment. OSA-associated CKD is mainly triggered by CIH-induced renal damage (Lin et al., 2018; Beaudin et al., 2022). OSA triggers a subthreshold wake-up reaction because of a temporary shortage of oxygen to the various organs. Accumulating evidence has demonstrated that CKD is a relatively frequent complication in subjects with OSA. Recent studies suggested that the prevalence of OSA was significantly increased as renal function decreased, and the treatment of CPAP may be beneficial for kidney function among individuals with OSA (Voulgaris et al., 2019). CIH, a key characteristic of OSA, might result in renal disease *via* its potential association with inflammation, increased sympathetic nervous system activity, activation of the renin–angiotensin–aldosterone system, intrarenal hypoxia, and oxidative stress (Turek et al., 2012; Hanly and Ahmed, 2014; Lu et al., 2017). However, the exact underlying mechanism of OSA-related renal injury remains unclear.

miRNAs have attracted increasing attention because of their universality in gene regulation and their stability among different samples. They are small noncoding RNA molecules less than 22 nucleotides in length. Moreover, miRNAs are important mediators of several pathological and physiological processes and have been found to affect various diseases. A previous study suggested that miRNAs may represent one of the promising therapeutic targets to treat inflammatory diseases in clinical practice (Lu et al., 2019). Liu et al. found that miR-132–3p could regulate osteoblast differentiation by repressing Smad5 in MC3T3-E1 osteoblastic cells in response to cyclic tensile stress (Liu et al., 2019). Additionally, it was reported that miR-374b could promote neural stem cell differentiation and proliferation *via* binding to Hes1 (Wu et al., 2018). However, the role of miRNAs in the pathophysiology of OSA-related CKD has not yet been described. Therefore, we applied our previously described CIH mouse model to reveal the underlying mechanisms of miRNAs under the renal injury induced by CIH.

In this study, 18 miRNAs were found to be differentially expressed in the process of CIH-induced kidney damage. Of these DEmiRs, nine miRNAs were upregulated and nine were downregulated. The eight selected DEmiRs were assessed using RT-qPCR, and the results of RT-qPCR were in accordance with the miRNA-seq data. These identified miRNAs were further found to be involved in a variety of biological and pathological processes. For instance, miR-212–3p in HGSOC inhibited cell invasion, proliferation, and migration by targeting MAP3K3 in high-grade serous ovarian cancer (Zhang et al., 2020). Additionally, miR-215–5p inhibition attenuated doxorubicin-induced HL-1 cell apoptosis and death by upregulating ZEB2 expression (Xiong et al., 2021). Furthermore, miR-96–5p could promote cell migration in breast cancer by activation of the MEK/ERK signaling pathway (Qin et al., 2020). A previous study also revealed that miR-96–5p downregulation might be used as a novel therapeutic strategy for treating allergic rhinitis (Zhan et al., 2021). However, the roles of these DEmiRs in the development of CIH-induced renal injury remain unclear.

To further reveal the biological function of these screened miRNAs in the progress of renal damage caused by CIH, GO and KEGG enrichment analyses were carried out. Our findings showed that 141 pathways might be regulated by these DEmiRs. For the upregulated DEmiRs, the Wnt signaling pathway was the most enriched one. Xiong et al. (2020) reported that the Wnt/β-catenin pathway was related with hypoxia-related factors (HIFs). Meanwhile, it has been demonstrated that HIFs are very relevant in patients with OSA (Prabhakar et al., 2020). In addition, it was suggested that the Wnt/β-catenin signaling pathway was implicated in the development of CIH-induced cognitive impairment (Pan et al., 2016). Previous studies have also found that hypoxic conditions might activate Wnt/β-catenin pathways through DKK1 inhibition (Wang et al., 2022) and GSK3β inactivation (Mayes et al., 2011). One latest study explored that CIH could aggravate the process of skeletal muscle aging by inhibiting KLC1/GRX1 expression through the Wnt/β-catenin pathway (Guo et al., 2021). Thus, even though it was demonstrated that Wnt/β-catenin was a key signaling pathway concerning OSA, the exact mechanism in CIH-induced kidney injury was not well understood.

Since the prominent biological function of miRNAs is to negatively regulate downstream mRNAs by targeting their 3′-untranslated region, we then predicted putative targets of these DEmiRs using two databases: miRWalk and miRDB. Through the complicated miRNA–mRNA network analysis, we discovered that one miRNA controlled a series of target genes, and numerous target genes were modulated by more than one miRNA. Elucidating the function of these targeted genes (e.g., Cep85L and EREG) was useful to enrich the current understanding of the complex molecular basis of renal damage induced by CIH. Meanwhile, erythropoietin (EPO), a small signaling molecule, was produced by the kidneys. Andrade et al. (2018) found that EPO was very relevant for patients affected by OSA. Thus, up/down-regulation of gene encoding EPO may be involved in the process of CIH-induced renal injury. Further research is in urgently needed to clarify the functions and molecular mechanisms of all these differentially expressed miRNAs.

However, limitations were present in the study. First, no cell-specific identification of these miRNAs was conducted, and how these miRNAs function in CIH-associated renal injury remained unclear. This should be the subject of further investigations. Second, our investigation is only limited to male mice. Whether gender plays any significant role in altered miRNA expression of CIH-induced kidney injury remains to be studied. Third, our research falls short of exposing mice to acute hypoxia for 4 h and measuring the expression profile of miRNAs. This will reduce the persuasiveness of the study. Fourth, we only use HE staining to verify the pathological damage of CIH. It is better to report the oxygen concentration in a cellular environment, the partial pressure of oxygen and partial pressure of carbon dioxide in blood, and the pH of blood and the Handerson–Hasselbalch ratio together. Fifth, we did not observe the effects of hypoxia in the different phases of sleep. Sixth, it lacks relevant details for a clinical perspective, which is very important for a common disease like OSA. Last but not least, the sample size was too small making the generalizability of the present results difficult to achieve.

## Conclusion

In summary, the present study first demonstrated the profiling of DEmiRs in a mouse model of kidney injury induced by CIH using miRNA-seq and revealed their functional interaction network. These findings expanded the current understanding of the molecular etiology of OSA-associated CKD, which may contribute to providing new therapeutic strategies for this disease.

## Data Availability

The data presented in the study are deposited in the GEO repository, accession number GSE202480.

## References

[B1] Andrade D. C. Haine L. Toledo C. Diaz H. S. Quintanilla R. A. Marcus N. J. (2018). Ventilatory and autonomic regulation in sleep apnea syndrome: A potential protective role for erythropoietin? Front. Physiol. 9, 1440. 10.3389/fphys.2018.01440 30374309 PMC6196773

[B2] Badran M. Abuyassin B. Ayas N. Sin D. D. Laher I. (2021). Vascular and renal telomere shortening in mice exposed to chronic intermittent hypoxia. Can. J. Physiol. Pharmacol. 99, 1112–1113. 10.1139/cjpp-2021-0143 33951396

[B3] Beaudin A. E. Raneri J. K. Ahmed S. B. Hirsch Allen A. J. M. Nocon A. Gomes T. (2022). Association of insomnia and short sleep duration, alone or with comorbid obstructive sleep apnea, and the risk of chronic kidney disease. Sleep 45, zsac088. 10.1093/sleep/zsac088 35445715 PMC9272259

[B4] Guo H. Zhang Y. Han T. Cui X. Lu X. (2021). Chronic intermittent hypoxia aggravates skeletal muscle aging by down-regulating Klc1/grx1 expression via Wnt/β-catenin pathway. Arch. Gerontol. Geriatr. 96, 104460. 10.1016/j.archger.2021.104460 34218156

[B5] Hanly P. J. Ahmed S. B. (2014). Sleep apnea and the kidney. Chest 146, 1114–1122. 10.1378/chest.14-0596 25288001

[B6] Lai S. Chen L. Zhan P. Lin G. Lin H. Huang H. (2021). Circular RNA expression profiles and bioinformatic analysis in mouse models of obstructive sleep Apnea-Induced cardiac injury: Novel insights into pathogenesis. Front. Cell Dev. Biol. 9, 767283. 10.3389/fcell.2021.767283 34820383 PMC8606653

[B7] Light M. McCowen K. Malhotra A. Mesarwi O. A. (2018). Sleep apnea, metabolic disease, and the cutting edge of therapy. Metabolism. 84, 94–98. 10.1016/j.metabol.2017.09.004 28966076 PMC5874161

[B8] Liguori C. Maestri M. Spanetta M. Placidi F. Bonanni E. Mercuri N. B. (2021). Sleep-disordered breathing and the risk of Alzheimer's disease. Sleep. Med. Rev. 55, 101375. 10.1016/j.smrv.2020.101375 33022476

[B9] Lin C. Lurie R. C. Lyons O. D. (2020). Sleep apnea and chronic kidney disease: A state-of-the-art review. Chest 157, 673–685. 10.1016/j.chest.2019.09.004 31542452

[B10] Lin C. Perger E. Lyons O. D. (2018). Obstructive sleep apnea and chronic kidney disease. Curr. Opin. Pulm. Med. 24, 549–554. 10.1097/MCP.0000000000000525 30239379

[B11] Liu M. Sun F. Feng Y. Sun X. Li J. Fan Q. (2019). MicroRNA-132-3p represses Smad5 in MC3T3-E1 osteoblastic cells under cyclic tensile stress. Mol. Cell. Biochem. 458, 143–157. 10.1007/s11010-019-03538-3 31004309

[B12] Lu Q. Wu R. Zhao M. Garcia-Gomez A. Ballestar E. (2019). MiRNAs as therapeutic targets in inflammatory disease. Trends Pharmacol. Sci. 40, 853–865. 10.1016/j.tips.2019.09.007 31662207

[B13] Lu W. Kang J. Hu K. Tang S. Zhou X. Yu S. (2017). Angiotensin-(1-7) relieved renal injury induced by chronic intermittent hypoxia in rats by reducing inflammation, oxidative stress and fibrosis. Braz J. Med. Biol. Res. 50, e5594. 10.1590/1414-431X20165594 28076452 PMC5264539

[B14] Matsuyama H. Suzuki H. I. (2020). Systems and synthetic microRNA biology: From biogenesis to disease pathogenesis. Int. J. Mol. Sci. 21, 132. 10.3390/ijms21010132 PMC6981965 31878193

[B15] Mayes P. A. Dolloff N. G. Daniel C. J. Liu J. J. Hart L. S. Kuribayashi K. (2011). Overcoming Hypoxia-Induced apoptotic resistance through combinatorial inhibition of GSK-3β and CDK1. Cancer Res. 71, 5265–5275. 10.1158/0008-5472.CAN-11-1383 21646472 PMC3667402

[B16] Mesarwi O. A. Loomba R. Malhotra A. (2019). Obstructive sleep apnea, hypoxia, and nonalcoholic fatty liver disease. Am. J. Respir. Crit. Care Med. 199, 830–841. 10.1164/rccm.201806-1109TR 30422676 PMC6835083

[B17] Nguyen D. Chilian W. M. Zain S. M. Daud M. F. Pung Y. F. (2021). MicroRNA regulation of vascular smooth muscle cells and its significance in cardiovascular diseases. Can. J. Physiol. Pharmacol. 99, 827–838. 10.1139/cjpp-2020-0581 33529092

[B18] Nowinski A. Czyzak-Gradkowska A. Jonczak L. Korzybski D. Peradzynska J. Plywaczewski R. (2020). Glomerular filtration rate in patients with obstructive sleep apnea: The influence of cystatin-C-based estimations and comorbidity. J. Thorac. Dis. 12, 175–183. 10.21037/jtd.2020.02.11 32274082 PMC7139095

[B19] Pan H. W. Li S. C. Tsai K. W. (2013). MicroRNA dysregulation in gastric cancer. Curr. Pharm. Des. 19, 1273–1284. 10.2174/138161213804805621 23092346

[B20] Pan Y. Deng Y. Xie S. Wang Z. Wang Y. Ren J. (2016). Altered Wnt signaling pathway in cognitive impairment caused by chronic intermittent hypoxia: Focus on glycogen synthase kinase-3β and β-catenin. Chin. Med. J. 129, 838–845. 10.4103/0366-6999.178969 26996481 PMC4819306

[B21] Prabhakar N. R. Peng Y. Nanduri J. (2020). Hypoxia-inducible factors and obstructive sleep apnea. J. Clin. Invest. 130, 5042–5051. 10.1172/JCI137560 32730232 PMC7524484

[B22] Qin W. Y. Feng S. C. Sun Y. Q. Jiang G. Q. (2020). MiR‐96‐5p promotes breast cancer migration by activating MEK/ERK signaling. J. Gene Med. 22, e3188. 10.1002/jgm.3188 32196830

[B23] Schütz S. G. Dunn A. Braley T. J. Pitt B. Shelgikar A. V. (2021). New frontiers in pharmacologic obstructive sleep apnea treatment: A narrative review. Sleep. Med. Rev. 57, 101473. 10.1016/j.smrv.2021.101473 33853035

[B24] Torres J. Novo-Veleiro I. Manzanedo L. Alvela-Suárez L. Macías R. Laso F. (2018). Role of microRNAs in alcohol-induced liver disorders and non-alcoholic fatty liver disease. World J. Gastroenterol. 24, 4104–4118. 10.3748/wjg.v24.i36.4104 30271077 PMC6158486

[B25] Turek N. F. Ricardo A. C. Lash J. P. (2012). Sleep disturbances as nontraditional risk factors for development and progression of CKD: Review of the evidence. Am. J. Kidney Dis. 60, 823–833. 10.1053/j.ajkd.2012.04.027 22727724 PMC3461247

[B26] Voulgaris A. Marrone O. Bonsignore M. R. Steiropoulos P. (2019). Chronic kidney disease in patients with obstructive sleep apnea. A narrative review. Sleep. Med. Rev. 47, 74–89. 10.1016/j.smrv.2019.07.001 31376590

[B27] Wang X. He Y. Mackowiak B. Gao B. (2021). MicroRNAs as regulators, biomarkers and therapeutic targets in liver diseases. Gut 70, 784–795. 10.1136/gutjnl-2020-322526 33127832

[B28] Wang D. Liu Z. Yan Z. Liang X. Liu X. Liu Y. (2021). MiRNA-155-5p inhibits epithelium-to-mesenchymal transition (EMT) by targeting GSK-3β during radiation-induced pulmonary fibrosis. Arch. Biochem. Biophys. 697, 108699. 10.1016/j.abb.2020.108699 33259794

[B29] Wang Q. Tian J. Li X. Liu X. Zheng T. Zhao Y. (2022). Upregulation of endothelial DKK1 (Dickkopf 1) promotes the development of pulmonary hypertension through the sp1 (Specificity protein 1)/SHMT2 (Serine hydroxymethyltransferase 2) pathway. Hypertension 79, 960–973. 10.1161/HYPERTENSIONAHA.121.18672 35249365

[B30] Wu X. Zhao X. Miao X. (2018). MicroRNA-374b promotes the proliferation and differentiation of neural stem cells through targeting Hes1. Biochem. Biophys. Res. Commun. 503, 593–599. 10.1016/j.bbrc.2018.06.044 29902458

[B31] Xiong Q. Liu B. Ding M. Zhou J. Yang C. Chen Y. (2020). Hypoxia and cancer related pathology. Cancer Lett. 486, 1–7. 10.1016/j.canlet.2020.05.002 32439418

[B32] Xiong X. He Q. Liu J. Dai R. Zhang H. Cao Z. (2021). MicroRNA miR-215-5p regulates doxorubicin-induced cardiomyocyte injury by targeting ZEB2. J. Cardiovasc. Pharmacol. 78, 622–629. 10.1097/FJC.0000000000001110 34282068

[B33] Yeghiazarians Y. Jneid H. Tietjens J. R. Redline S. Brown D. L. El-Sherif N. (2021). Obstructive sleep apnea and cardiovascular disease: A scientific statement from the American heart association. Circulation 144, e56–e67. 10.1161/CIR.0000000000000988 34148375

[B34] Zhan J. Zheng J. Zeng L. Fu Z. Huang Q. Wei X. (2021). Downregulation of miR-96-5p inhibits mTOR/NF-κb signaling pathway via DEPTOR in allergic rhinitis. Int. Arch. Allergy Immunol. 182, 210–219. 10.1159/000509403 33477144

[B35] Zhang L. Zhang Y. Wang S. Tao L. Pang L. Fu R. (2020). MiR-212-3p suppresses high-grade serous ovarian cancer progression by directly targeting MAP3K3. Am. J. Transl. Res. 12, 875–888. 32269720 PMC7137041

